# Predictors of Shoulder Pathology in Rheumatoid Arthritis: A Cross-Sectional Study

**DOI:** 10.7759/cureus.100215

**Published:** 2025-12-27

**Authors:** Raj Kumar, Ganesh Kumar, Abhinav Singh, Md Jawed Akhtar

**Affiliations:** 1 Department of Physical Medicine and Rehabilitation, Indira Gandhi Institute of Medical Sciences, Patna, IND; 2 Department of Physical Medicine and Rehabilitation, Employees State Insurance Corporation (ESIC) Medical College and Hospital (ESICMCH), Patna, IND; 3 Department of Anatomy, Indira Gandhi Institute of Medical Sciences, Patna, IND

**Keywords:** disability, hemoglobin, high-resolution musculoskeletal ultrasound, platelets, rheumatoid arthritis, seropositivity, shoulder pain, spadi, vitamin d

## Abstract

Background: Shoulder pain is a frequent but under‑recognized manifestation of rheumatoid arthritis (RA), contributing substantially to disability and impaired quality of life. High‑resolution musculoskeletal ultrasound (HRUS) offers sensitive detection of structural abnormalities, while the Shoulder Pain and Disability Index (SPADI) provides patient‑reported assessment of pain and function. However, the clinical and laboratory predictors of shoulder pathology and disability in RA remain incompletely defined.

Methods: In this cross‑sectional study, 105 RA patients presenting with shoulder pain were enrolled. All participants underwent HRUS evaluation of the affected shoulder(s) and completed the SPADI questionnaire. Baseline demographic and laboratory parameters were recorded. Binary logistic regression was used to identify predictors of abnormal HRUS findings and seropositivity, while linear regression analyses examined associations with SPADI total (SPADI‑T), pain (SPADI‑P), and disability (SPADI‑D) scores.

Results: Abnormal HRUS findings were present in 69 patients (65.7%). Logistic regression identified lower Hb (p=0.006, OR=0.01) and higher platelet (PLT) count (p=0.016, OR=1.16) as significant predictors of abnormal HRUS findings, with erythrocyte sedimentation rate (ESR) (p=0.010) and vitamin D deficiency (p=0.050) approaching significance. Higher ESR was also correlated with higher CRP. Linear regression demonstrated that longer disease duration, lower Hb, higher PLT count, elevated random blood sugar, and higher thyroid-stimulating hormone were predictors of worse SPADI‑T scores (R²=54.4%).

Conclusion: Shoulder pathology in RA is multifactorial, with anemia, thrombocytosis, systemic inflammation, metabolic derangements, and vitamin D deficiency contributing to pain and disability. HRUS abnormalities are strongly linked to hematologic and metabolic markers, while functional impairment is influenced by both inflammatory and endocrine factors.

## Introduction

Rheumatoid arthritis (RA) is a chronic, systemic autoimmune disease characterized by persistent synovial inflammation, progressive joint destruction, and extra‑articular manifestations that collectively contribute to significant morbidity and impaired quality of life [1]. Among the various joints affected, the shoulder occupies a unique position due to its complex anatomy and pivotal role in upper limb function. Shoulder involvement in RA is often under‑recognized compared to small joints of the hands and feet, yet it can substantially limit daily activities, compromise independence, and reduce overall functional capacity. Pain, stiffness, and disability arising from shoulder pathology in RA patients therefore represent an important clinical burden that warrants systematic evaluation [2-4].

The shoulder joint is particularly vulnerable in RA because of its synovial lining, large capsular surface, and dependence on periarticular soft tissues for stability. Inflammatory processes can lead to synovitis, bursitis, rotator cuff tendinopathy, and erosive changes in the glenohumeral and acromioclavicular joints. These pathologies manifest clinically as pain and restricted range of motion, often progressing insidiously and contributing to cumulative disability [5,6]. Despite the clinical relevance, shoulder involvement in RA has historically received less attention in research compared to peripheral joints, resulting in gaps in understanding of its predictors and correlates.

High‑resolution ultrasonography (HRUS) has emerged as a sensitive, non‑invasive imaging modality for detecting early inflammatory and structural changes in RA. HRUS can visualize synovial hypertrophy, effusion, erosions, and tendon abnormalities with greater sensitivity than conventional radiography, and without the radiation exposure associated with computed tomography [7,8]. In the context of shoulder pathology, HRUS provides valuable insights into both intra‑articular and periarticular structures, enabling clinicians to identify abnormalities that may not be apparent on clinical examination alone [9,10]. However, the clinical and laboratory predictors of abnormal HRUS findings in RA shoulders remain incompletely defined.

Parallel to imaging, patient‑reported outcome measures are essential for capturing the subjective burden of disease. The Shoulder Pain and Disability Index (SPADI) is a validated instrument that quantifies pain and functional limitation specific to the shoulder. It comprises two subscales, pain (SPADI‑P) and disability (SPADI‑D), which together yield a total score (SPADI‑T). SPADI has been widely used in musculoskeletal research and clinical practice, offering a patient‑centered perspective that complements objective imaging and laboratory assessments [5,11]. In RA, SPADI scores can provide a direct measure of how shoulder pathology impacts daily living, yet the determinants of higher SPADI scores in this population are not fully elucidated.

Several demographic and laboratory parameters have been implicated in RA disease activity and outcomes. Hematologic indices such as hemoglobin (Hb) and platelet (PLT) counts reflect systemic inflammation and anemia of chronic disease, both common in RA [12]. Elevated erythrocyte sedimentation rate (ESR) and total leukocyte count (TLC) serve as markers of inflammatory activity, while metabolic and endocrine parameters may influence musculoskeletal health through mechanisms of glycation, metabolic stress, and hormonal imbalance [12,13]. Despite these associations, few studies have comprehensively examined how these diverse parameters interact to predict both imaging abnormalities and patient‑reported shoulder disability in RA.

The present study was therefore designed to investigate predictors of shoulder pathology in RA using a cross‑sectional cohort of patients presenting with shoulder pain. Specifically, we aimed to correlate baseline demographic and laboratory parameters with HRUS findings and SPADI scores, including their pain and disability components. By employing regression analyses, we sought to delineate independent predictors of imaging abnormalities and patient‑reported outcomes, thereby advancing understanding of the multifactorial determinants of shoulder involvement in RA. Through this approach, we hope to highlight clinically relevant factors that extend beyond traditional rheumatologic markers, underscoring the need for comprehensive patient evaluation and multidisciplinary management in RA.

## Materials and methods

Study design and setting

This was a hospital‑based, cross‑sectional observational study conducted in the Department of Physical Medicine and Rehabilitation (PMR) at a tertiary care center in eastern India from January 2025 to June 2025. The study was carried out over a six‑month period following approval from the Institutional Ethics Committee. Written informed consent was obtained from all participants prior to enrollment.

Participants

Adult patients (≥18 years) diagnosed with RA according to the 2010 American College of Rheumatology/European League Against Rheumatism (ACR/EULAR) criteria were consecutively recruited [14]. Inclusion criteria were RA patients presenting with unilateral or bilateral shoulder pain of at least two weeks’ duration. Patients with functional shoulder pain, non‑organic symptoms, psychiatric illness interfering with reliable history, or referred pain from cervical radiculopathy were excluded after clinical evaluation.

A total of 105 RA patients with shoulder pain were enrolled. A sample size of 105 was calculated (assuming 40% prevalence, 95% confidence interval [5], 10% absolute precision, and 10% attrition rate). The calculation was performed by using the standard formula in which the sample size (n) was equal to the square of the Z‑value multiplied by the prevalence (p) multiplied by one minus the prevalence (1 - p), and the entire product is divided by the square of the desired absolute precision (d²):

$n = \frac{Z^{2} \times p \times (1 - p)}{d^{2}}$

Demographic details (age, sex, disease duration) and baseline laboratory parameters were recorded, including hemoglobin (Hb), total leukocyte count (TLC), neutrophil and lymphocyte counts, PLT count, erythrocyte sedimentation rate (ESR), random blood sugar (RBS), thyroid‑stimulating hormone (TSH), vitamin D levels, and liver enzymes (SGOT, SGPT). Serological status (rheumatoid factor and anti‑cyclic citrullinated peptide antibodies) was documented to classify patients as seropositive or seronegative.

Clinical assessment

Shoulder pain and functional disability were assessed using the Shoulder Pain and Disability Index (SPADI), a validated patient‑reported outcome measure. The SPADI consists of 13 items divided into two subscales: Pain (SPADI‑P, 5 items) and Disability (SPADI‑D, eight items). Each item was scored on a 0-10 visual analog scale, with higher scores indicating greater severity. Subscale scores were averaged and expressed as percentages, and the total score (SPADI‑T) was calculated as the mean of pain and disability components [11].

Imaging assessment

All patients underwent high‑resolution musculoskeletal ultrasound (HRUS) of the affected shoulder(s). HRUS was performed by a trained physiatrist with over 10 years of experience, using a Fujifilm Sonosite M‑Turbo system equipped with a high‑frequency (6-15 MHz) linear array transducer. The examination followed the European Society of Skeletal Radiology (ESSR 2016) guidelines for standardized shoulder ultrasound [15]. Structures assessed included glenohumeral (GH) joint (effusion, synovitis, erosions); subacromial‑subdeltoid (SASD) and subcoracoid bursae; rotator cuff tendons (supraspinatus, infraspinatus, subscapularis) for tendinosis or tears; long head of biceps tendon (effusion, tendinitis, subluxation); acromioclavicular joint integrity.

Ultrasound abnormalities were defined using standardized criteria consistent with ESSR recommendations, including non‑compressible hypoechoic synovial thickening (>2 mm) for synovial hypertrophy, compressible anechoic/hypoechoic fluid for effusion, and bursal fluid >2 mm for bursitis. Tendinosis was identified by hypoechogenicity, tendon thickening, and loss of fibrillar architecture, while partial‑ and full‑thickness tears were diagnosed by focal or complete tendon discontinuity visualized in two orthogonal planes. Biceps pathology included sheath fluid >2 mm for tenosynovitis, tendon thickening/hypoechogenicity for tendinitis, and medial displacement on dynamic scanning for subluxation. Cortical step‑down defects in two planes defined erosions, and dynamic bursal bunching during abduction indicated impingement.

Dynamic impingement was evaluated using ultrasound‑adapted Neer and Hawkins‑Kennedy maneuvers during active abduction and internal rotation. HRUS findings were recorded as categorical variables (present/absent).

Statistical analysis

Data were analyzed using Minitab v21 and IBM SPSS Statistics for Windows, Version 25 (Released 2017; IBM Corp., Armonk, New York, United States). Descriptive statistics (mean ± SD, median with interquartile range, percentages) were used to summarize demographic, laboratory, and imaging data. Binary logistic regression was performed to identify predictors of abnormal HRUS findings and seropositivity, with odds ratios (ORs) and 95% confidence intervals (CIs) reported. Linear regression analyses were conducted to evaluate associations between baseline parameters and SPADI scores (SPADI‑T, SPADI‑P, SPADI‑D). Regression coefficients, standard errors, t‑values, and p‑values were calculated. Model fit was assessed using R‑squared and adjusted R‑squared values. Multicollinearity was evaluated using variance inflation factors (VIFs), and all included variables demonstrated VIF values below accepted thresholds, indicating no significant multicollinearity. A p‑value <0.05 was considered statistically significant.

## Results

One hundred and five patients with RA and shoulder pain were enrolled in our study with the objective of correlating baseline demographic and lab parameters with SPADI with components (P for pain, D for disability, and T for total). Of these 105 patients, 69 patients (65.7%) had abnormalities in HRUS findings, and 36 patients (34.3 %) had no HRUS findings. SASD bursitis was the most common abnormality, followed by subcoracoid bursitis. Less frequent findings included synovitis, biceps tendon effusion, GH effusion, arthritis, and erosion, while tear generation/tendinosis was rare.

Significant differences were observed in hemoglobin, PLT count, ESR, random blood sugar (RBS), TSH, vitamin D, and SPADI-T scores, all showing p-values <0.05, indicating worse inflammatory and metabolic profiles in the HRUS-positive group. Parameters like age, TLC, SGOT, SGPT, and lymphocyte percentage showed no significant difference, suggesting that HRUS findings are more closely associated with specific inflammatory and endocrine markers rather than general hematologic or hepatic indices (Table 1).

**Table 1:** Comparison of baseline demographic, clinical, and laboratory variables TLC: Total leukocyte count; NEU: Neutrophil; SPADI-T: Shoulder Pain and Disability Index total; SGPT: Serum glutamate pyruvate transaminase; SGOT: Serum glutamic-oxaloacetic transaminase; RBS: Random blood sugar; ESR: Erythrocyte sedimentation rate

Parameters	Parameter Value (Mean ± SD)		T-Value	P-Value (Unpaired t Test)
	With HRUS Finding (n=69)	Without HRUS Finding (n=36)		
Age in Years	44.94 ± 13.46	44.58 ± 13.83	0.1289	0.8977
Hb in g/dl	10.22 ± 1.44	12.66 ± 1.34	8.436	<0.0001
TLC in 1000/µL	9.81 ± 3.08	9.9 ± 3.3	0.1387	0.89
NEU in %	68.39 ± 8.66	63.09 ± 12.57	2.538	0.0127
LYM %	22.63 ± 8.39	25.62 ± 8.47	1.728	0.087
PLT %	409.28 ± 150.25	197.53 ± 53.75	8.172	<0.0001
ESR in mm/hr	66.41 ± 26.43	29.11 ± 16.61	7.701	<0.0001
RBS in mg/dl	124.76 ± 53.65	88 ± 17.69	3.991	0.0001
TSH in µIU/mL	4.41 ± 5.08	1.48 ± 1.2	3.404	0.0009
Vit D in ng/dl	16.36 ± 5.15	24.67 ± 10.72	5.374	<0.0001
SGOT in U/L	47.56 ± 44.53	43.89 ± 29.17	0.4465	0.6562
SGPT in U/L	42.61 ± 32.15	41.45 ± 28.17	0.1829	0.8553
SPADI-T Score	36.1 ± 8.51	26.62 ± 10.7	4.952	<0.0001

The results show that lower Hb levels and higher PLT counts are statistically significant predictors, as indicated by their p-values (0.006 and 0.016, respectively). Specifically, for every 1g/dl change in Hb, the odds of having a positive HRUS finding decrease markedly (OR: 0.01), while higher PLT counts are associated with slightly increased odds (OR: 1.16). ESR and Vitamin D also approach significance (p=0.010 and p=0.050), with higher ESR and lower Vitamin D potentially increasing the likelihood of an abnormal ultrasound (Table 2).

**Table 2:** Binary logistic regression analysis of positive HRUS findings with baseline demographic and lab parameters TLC: Total leukocyte count; NEU: Neutrophil; SPADI-T: Shoulder Pain and Disability Index total; SGPT: Serum glutamate pyruvate transaminase; SGOT: Serum glutamic-oxaloacetic transaminase; RBS: Random blood sugar; ESR: Erythrocyte sedimentation rate

Predictor	Coef	SE Coef	Z	P	Odds Ratio	95% CI Lower	95% CI Upper
Constant	21.6580	28.7320	1.03	0.302	–	–	–
Age	0.0954339	0.0707485	1.35	0.177	1.10	0.96	1.26
Hb	-4.82179	1.74799	-2.76	0.006	0.01	0.00	0.25
TLC	-0.439586	0.337605	-1.30	0.193	0.64	0.33	1.25
NEU	0.396663	0.212170	1.87	0.062	1.49	0.98	2.25
LYM	-0.002607	0.269624	-0.01	0.992	1.00	0.59	1.69
PLT	0.145094	0.0604333	2.40	0.016	1.16	1.03	1.30
ESR*	0.361350	0.140811	2.57	0.010	1.11	0.53	1.92
RBS	0.0479069	0.0585439	0.82	0.413	1.05	0.94	1.18
TSH	1.45967	0.941863	1.55	0.121	0.23	0.04	1.47
Vit D	-0.693292	0.353040	-1.96	0.050	0.50	0.25	1.00
SGOT	0.0510298	0.0438503	1.16	0.245	1.05	0.97	1.15
SGPT	0.007183	0.0432652	0.17	0.868	0.99	0.91	1.08
SPADI-T	0.353470	0.165585	2.13	0.033	1.12	0.51	0.97

**Figure 1:**
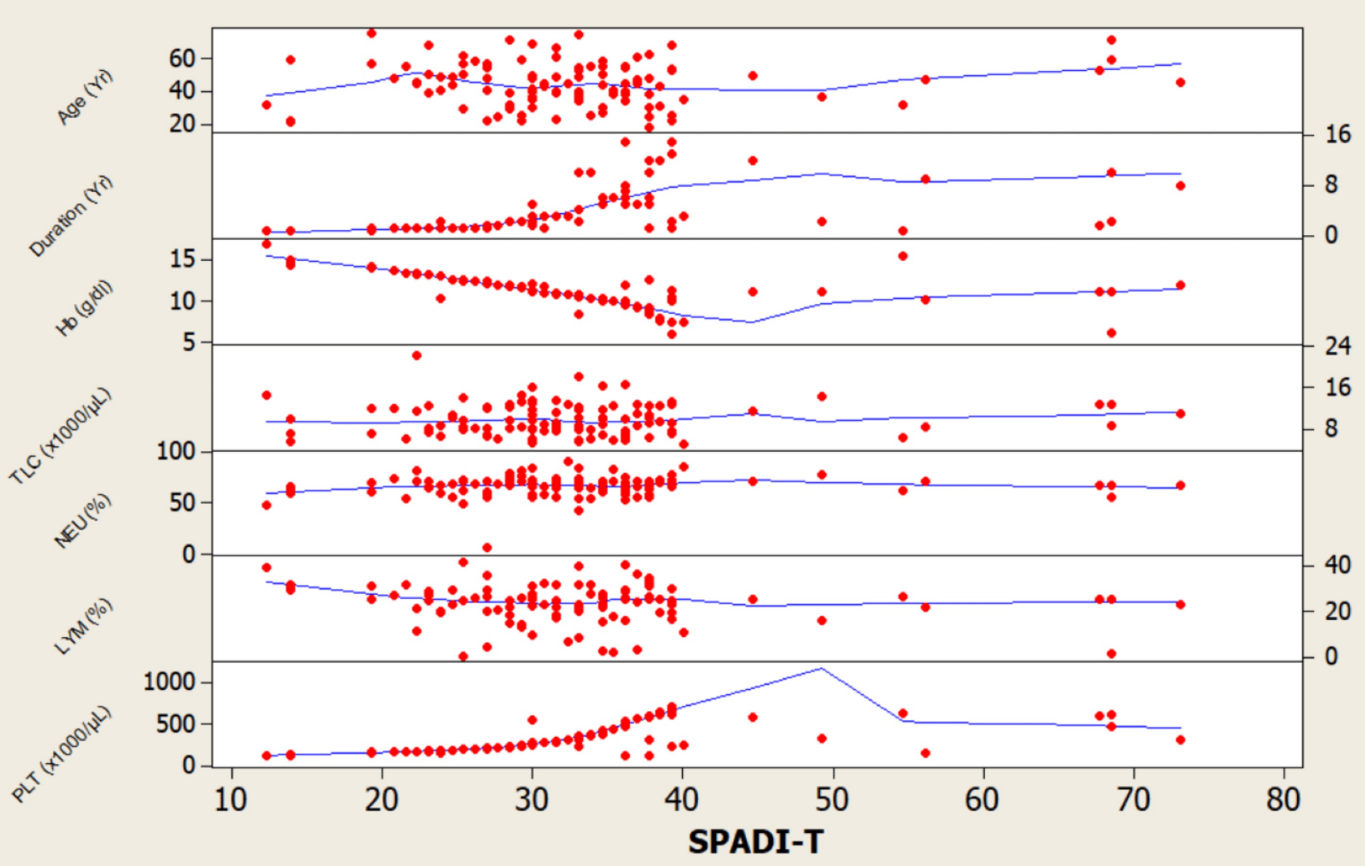
Scatterplot matrix illustrating the bivariate relationships between SPADI‑T scores and key demographic and hematologic variables Hb: Hemoglobin; PLT: Platelet; LYM: Lymphocyte; NEU: Neutrophil; SPADI-T: Shoulder Pain and Disability Index-total. Each red data point corresponds to a patient.

**Figure 2:**
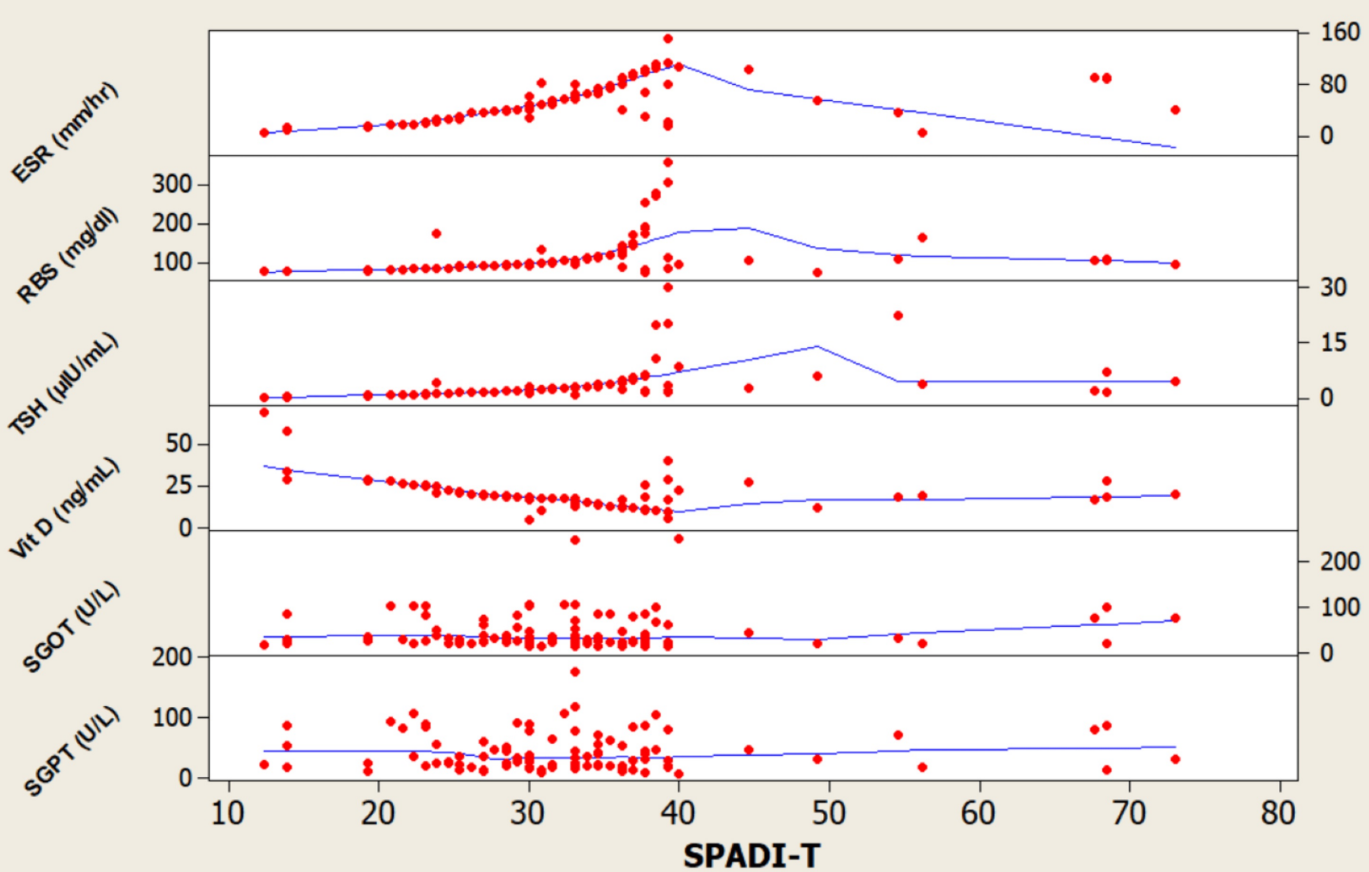
Scatterplot matrix showing the bivariate relationships between SPADI‑T scores and inflammatory, metabolic, endocrine, and hepatic parameters Hb: Hemoglobin; PLT: Platelet; LYM: Lymphocyte; NEU: Neutrophil; SPADI-T: Shoulder Pain and Disability Index-total Each red data point corresponds to a patient.

The model is significant, explaining 54.4% of the variance in SPADI-T scores. It identifies several independent predictors of worse total disability: longer disease duration, lower Hb, higher PLT count, higher RBS, and higher TSH. This suggests that a patient's overall shoulder pain and disability is significantly influenced not just by rheumatologic factors, but also by anemia, metabolic, and endocrine status (Table 3, Figure 1, Figure 2).

**Table 3:** Linear regression analysis of SPADI-T (SPADI-P + SPADI-D) with baseline demographic and lab parameters The regression equation is: SPADI-T = 47.6 + 0.0423 Age + 0.809 Duration - 2.92 Hb + 0.262 TLC + 0.0264 NEU + 0.023 LYM + 0.0342 PLT + 0.0787 ESR + 0.174 RBS + 1.03 TSH - 0.020 Vit D + 0.0058 SGOT + 0.0028 SGPT S = 7.44387   R-Sq = 54.4%   R-Sq(adj) = 47.9% TLC: Total leukocyte count; NEU: Neutrophil; LYM: Lymphocyte

Predictor	Coef	SE Coef	T	P
Constant	47.60	14.10	4.79	<0.0001
Age	0.04228	0.05532	0.76	0.447
Duration	0.8087	0.2995	2.70	0.008
Hb	-2.9191	0.9621	-3.03	0.003
TLC	0.2620	0.2666	0.98	0.328
NEU	0.02637	0.08220	0.32	0.749
LYM	0.0230	0.1083	0.21	0.832
PLT	0.034183	0.008714	3.92	<0.0001
ESR	0.07870	0.06450	1.22	0.226
RBS	0.17373	0.03469	5.01	<0.0001
TSH	1.0253	0.3061	3.35	0.001
Vit D	-0.0199	0.1200	-0.17	0.869
SGOT	0.00579	0.02792	0.21	0.836
SGPT	0.00277	0.03682	0.08	0.940

For the pain component of the SPADI score, Table 4 explains 48.5% of the variance and reveals a distinct set of significant predictors compared to the total score. Higher pain scores are significantly associated with lower hemoglobin (Hb), higher TLC, higher lymphocyte count (LYM), higher PLT, higher RBS, and lower vitamin D levels. This indicates that the experience of pain is independently linked to anemia, systemic inflammation (as suggested by TLC, LYM, and PLT), glycemic state, and vitamin D deficiency.

**Table 4:** Linear regression analysis of SPADI-P with baseline demographic and lab parameters The regression equation is: SPADI-P = 42.1 + 0.0328 Age + 0.051 Duration - 2.65 Hb + 1.04 TLC + 0.0199 NEU + 0.270 LYM + 0.0223 PLT + 0.0275 ESR + 0.0985 RBS + 0.554 TSH - 0.320 Vit D + 0.0275 SGOT + 0.0673 SGPT S = 8.17963   R-Sq = 48.5%   R-Sq(adj) = 41.1% TLC: Total leukocyte count; NEU: Neutrophil; SGPT: Serum glutamate pyruvate transaminase; SGOT: Serum glutamic-oxaloacetic transaminase; RBS: Random blood sugar; ESR: Erythrocyte sedimentation rate

Predictor	Coef	SE Coef	T	P
Constant	42.11	15.49	4.01	0.000
Age	0.03277	0.06079	0.54	0.591
Duration	0.0512	0.3291	0.16	0.877
Hb	-2.649	1.057	-2.51	0.014
TLC	1.0449	0.2930	3.57	0.001
NEU	0.01991	0.09033	0.22	0.826
LYM	0.2698	0.1191	2.27	0.026
PLT	0.022330	0.009576	2.33	0.022
ESR	0.02745	0.07088	0.39	0.699
RBS	0.09848	0.03812	2.58	0.011
TSH	0.5541	0.3363	1.65	0.103
Vit D	-0.3197	0.1319	-2.42	0.017
SGOT	0.02750	0.03068	0.90	0.372
SGPT	0.06734	0.04046	1.66	0.099

Higher TLC was positively associated with SPADI-D (coef 0.69, p=0.043), while increased PLT also showed a borderline positive effect (coef 0.02, p=0.050). In contrast, lower vitamin D levels were strongly linked to higher disability scores (Coef -0.50, p=0.001), underscoring its clinical relevance. Age, duration of illness, hemoglobin, neutrophils, lymphocytes, ESR, RBS, TSH, SGOT, and SGPT did not reach statistical significance, though SGPT trended toward association (p=0.080). Overall, the findings highlight that inflammatory markers (TLC, PLT) and vitamin D deficiency are key contributors to functional disability, suggesting their potential role in patient stratification and management (Table 5).

**Table 5:** Linear regression analysis of SPADI-D with baseline demographic and lab parameters The regression equation is: SPADI-D = 35.3 + 0.0955 Age + 0.159 Duration - 1.24 Hb + 0.687 TLC + 0.067 NEU + 0.157 LYM + 0.0218 PLT + 0.0411 ESR + 0.0678 RBS + 0.404 TSH - 0.500 Vit D + 0.0345 SGOT + 0.0820 SGPT S = 9.35740   R-Sq = 34.8%   R-Sq(adj) = 25.5% TLC: Total leukocyte count; NEU: Neutrophil; SGPT: Serum glutamate pyruvate transaminase; SGOT: Serum glutamic-oxaloacetic transaminase; RBS: Random blood sugar; ESR: Erythrocyte sedimentation rate

Predictor	Coef	SE Coef	T	P
Constant	35.32	17.73	3.18	0.002
Age	0.09548	0.06954	1.37	0.173
Duration	0.1592	0.3765	0.42	0.673
Hb	-1.241	1.209	-1.03	0.307
TLC	0.6874	0.3351	2.05	0.043
NEU	0.0669	0.1033	0.65	0.519
LYM	0.1567	0.1362	1.15	0.253
PLT	0.02175	0.01095	1.99	0.050
ESR	0.04111	0.08108	0.51	0.613
RBS	0.06783	0.04361	1.56	0.123
TSH	0.4043	0.3848	1.05	0.296
Vit D	-0.4999	0.1509	-3.31	0.001
SGOT	0.03446	0.03510	0.98	0.329
SGPT	0.08200	0.04628	1.77	0.080

Table 6 examines factors associated with RA seropositivity. Among all the demographic and lab parameters tested, only increasing age shows a statistically significant association with the likelihood of being seropositive (p=0.039). No other variables, including inflammatory markers, blood counts, or the SPADI-T score, demonstrated a significant relationship with seropositive status in this patient cohort, suggesting that seropositivity is largely independent of the other measured clinical and laboratory parameters in this study. This may be due to very small number of seronegative patients (n=7). 

**Table 6:** Binary logistic regression analysis of seropositivity with baseline demographic and lab parameters

Predictor	Coef	SE Coef	Z	p	Odds Ratio	95% CI Lower	95% CI Upper
Constant	-26.5797	39.8732	-0.49	0.623	–	–	–
Age	0.132914	0.0643539	2.07	0.039	1.19	0.77	0.99
Hb	1.87205	2.34386	0.80	0.424	6.50	0.07	642.91
TLC	0.874913	0.588143	1.49	0.137	2.40	0.76	7.60
NEU	0.390422	0.359582	1.09	0.278	0.68	0.33	1.37
LYM	0.103102	0.153205	0.67	0.501	1.11	0.82	1.50
PLT	0.0278167	0.0200772	1.39	0.166	1.03	0.99	1.07
ESR	0.132324	0.123439	1.07	0.284	1.14	0.90	1.45
RBS	0.376969	0.220433	1.71	0.087	1.46	0.95	2.25
TSH	1.04368	1.43170	0.73	0.466	2.84	0.17	46.98
Vit D	-0.454141	0.331065	-1.37	0.170	0.63	0.33	1.22
SGOT	0.0177408	0.0546732	0.32	0.746	1.02	0.91	1.13
SGPT	0.0012247	0.0458191	0.03	0.979	1.00	0.92	1.10
SPADI-T	0.465868	0.346789	1.34	0.179	1.05	0.32	1.24

## Discussion

This study investigates the multifaceted nature of shoulder pathology in patients with RA, moving beyond traditional rheumatologic markers to explore the influence of hematologic, metabolic, and endocrine factors.

The clinical significance is substantial. Shoulder involvement in RA is common and leads to significant functional impairment and reduced quality of life. Understanding which factors predict structural damage (via HRUS) and patient-reported outcomes (via SPADI) can guide more personalized and proactive management. For instance, identifying modifiable risk factors like anemia, hyperglycemia, or vitamin D deficiency opens up adjunctive therapeutic avenues beyond conventional disease-modifying antirheumatic drugs.

Lower Hb was a consistent and strong predictor across all analyses. Conversely, a higher PLT count was a significant predictor of abnormal HRUS (OR: 1.16, p=0.016) and worse SPADI-Total (p<0.0001) and SPADI-Pain (p=0.022) scores, with a borderline association to SPADI-Disability (p=0.050).

Anemia of chronic disease is a well-known extra-articular manifestation of RA, driven by pro-inflammatory cytokines like IL-6 that disrupt iron homeostasis and erythropoiesis. Thus, low Hb serves as a surrogate marker for more severe or systemic inflammatory burden, which directly translates to more joint damage and pain. PLTs are increasingly recognized as active participants in inflammation rather than passive bystanders. In RA, activated PLTs release pro-inflammatory mediators and can contribute to synovitis and vascular proliferation. The positive association of PLT with both ultrasound damage and disability scores underscores its role as a marker of ongoing inflammatory activity.

Our findings on PLTs are strongly supported by the work of Targońska-Stępniak et al. (2021), who found that PLT count and plateletcrit (PCT) correlated significantly with DAS28, CRP, ESR, and power Doppler ultrasound (PDUS) scores. They concluded that PLT indices are useful proxies for local and systemic inflammation [16]. The central role of inflammation in driving outcomes is also consistent with Bird et al. (2017), who identified lower disease activity at baseline as a key predictor of remission. While Bird et al. did not highlight Hb or PLT specifically, their findings align with the overarching principle that the systemic inflammatory milieu is a primary determinant of disease outcomes [17].

ESR was a significant predictor of abnormal HRUS (p=0.010). Higher ESR was also correlated with higher CRP. Higher RBS was strongly associated with worse SPADI-Total (p<0.0001) and SPADI-Pain (p=0.011) scores. An elevated TLC was linked to greater SPADI-Pain (p=0.001) and SPADI-Disability (p=0.043).

ESR's association with ultrasound abnormalities confirms its utility as a classic marker of systemic inflammation in RA. The strong link between RBS and worse shoulder disability is intriguing and points to a potential metabolic component in RA-related pain and dysfunction. Hyperglycemia can exacerbate systemic inflammation through the formation of advanced glycation end-products and oxidative stress, potentially worsening pain perception and functional limitation. The association of TLC, and specifically LYM, with pain reflects the fundamental role of cellular immune infiltration in the synovium, driving the local inflammatory process that directly causes pain.

The prognostic value of imaging-detected inflammation over clinical markers, as suggested by our HRUS findings, is powerfully echoed by Yonemoto et al. (2017). They found that baseline synovitis on PET/MRI was a superior predictor of long-term shoulder destruction compared to clinical activity scores and inflammatory markers. This reinforces the concept that subclinical inflammation, detectable only by imaging, is a critical driver of structural damage [18]. Furthermore, Alian et al. (2020) demonstrated a high prevalence (75%) of subclinical shoulder abnormalities on ultrasound in RA patients without shoulder symptoms, confirming that imaging reveals pathology that precedes clinical complaints [19].

Lower Vitamin D levels showed a borderline significant association with abnormal HRUS (p=0.050) and were strongly associated with higher SPADI-Disability scores (p=0.001). Higher TSH was a significant predictor of worse SPADI-Total scores (p=0.001). Vitamin D deficiency is prevalent in RA and has immunomodulatory roles. Deficiency is linked to increased disease activity and musculoskeletal pain, potentially through upregulation of pro-inflammatory cytokines and a direct effect on pain pathways. The strong link with disability may relate to vitamin D's role in muscle strength and function. The association with TSH suggests that subclinical or clinical hypothyroidism, which is common in autoimmune cohorts, may contribute to fatigue, myalgia, and overall functional impairment, compounding the disability from RA itself.

While the specific roles of Vitamin D and TSH were not highlighted in the cited studies, the finding that non-rheumatologic factors influence disability is consistent with the broader literature and the systematic review by Struyf et al. (2016). Their review found strong evidence that poor general health is a prognostic factor for the chronification of shoulder pain, which aligns with our findings that endocrine and metabolic health significantly impact the shoulder-specific disability measured by the SPADI [20].

The dissociation between seropositivity and shoulder-specific outcomes is clinically important. It suggests that once RA is established, the drivers of shoulder damage and disability may be more related to the current systemic inflammatory burden (reflected by Hb, PLT, TLC) and comorbid conditions (like hyperglycemia or vitamin D deficiency) than to the initial autoimmune serological profile.

This aligns with Bird et al. (2017), who found that RF and ACPA status were not significant predictors of remission [17]. Similarly, Boström et al. (1997) found that functional limitations and pain explained a large portion of disability variance, suggesting that the consequences of inflammation (like tendalgia and limited ROM) are more directly relevant to patient function than the serologic origin of the disease [21]. The importance of the SPADI score itself as a prognostic tool is strongly supported by Struyf et al. (2016), whose systematic review identified a high baseline SPADI score as one of the strongest predictors for the chronification of shoulder pain [20].

Some limitations of the study should also be considered while interpreting the results. As a cross-sectional study, the ability to infer causality is limited. The sample size of 105 though having enough statistical power was not large enough to detect all associations. There were only seven seronegative patients limiting the study's power to determine the association between seropositivity and other predictors. In the future, studies should be planned to compare HRUS findings and laboratory parameters between seropositive and seronegative patients with enough sample size.

## Conclusions

In this cross-sectional study of RA patients with shoulder pain, abnormal HRUS findings were common and were significantly predicted by hematologic and metabolic parameters, particularly lower hemoglobin and higher PLT counts, with ESR, CRP and vitamin D deficiency also contributing. Shoulder pain and disability (SPADI scores) were explained by a multifactorial model in which disease duration, anemia, thrombocytosis, hyperglycemia, and elevated TSH emerged as predictors of worse outcomes, while pain and disability components showed distinct associations with inflammatory markers and vitamin D status. Overall, these findings highlight the interplay of systemic inflammation, hematologic derangements, metabolic factors, and vitamin D deficiency in driving shoulder pathology and functional impairment in RA, suggesting that comprehensive management strategies should extend beyond rheumatologic control to address anemia, metabolic health, and endocrine balance.
